# Long non-coding RNA linc00152 acting as a promising oncogene in cancer progression

**DOI:** 10.5808/GI.2019.17.4.e36

**Published:** 2019-11-13

**Authors:** Danbi Seo, Dain Kim, Wanyeon Kim

**Affiliations:** 1 Department of Science Education, Korea National University of Education, Cheongju 28173, Korea; 2 Department of Biology Education, Korea National University of Education, Cheongju 28173, Korea

**Keywords:** linc00152, long non-coding RNA, oncogene, tumorigenesis

## Abstract

The incidence and mortality rate of cancer continues to gradually increase, although considerable research effort has been directed at elucidating the molecular mechanisms underlying biomarkers responsible for tumorigenesis. Accumulated evidence indicates that the long non-coding RNAs (lncRNAs), which are transcribed but not translated into functional proteins, contribute to cancer development. Recently, linc00152 (an lncRNA) was identified as a potent oncogene in various cancer types, and shown to be involved in cancer cell proliferation, invasiveness, and motility by sponging tumor-suppressive microRNAs acting as a competing endogenous RNA, binding to gene promoters acting as a transcriptional regulator, and binding to functional proteins. In this review, we focus on the oncogenic role of linc00152 in tumorigenesis and provided an overview of recent clinical studies on the effects of linc00152 expression in human cancers.

## Introduction

Cancers are diseases caused by abnormal cell growth. Unlike benign tumors where cells grow abnormally but do not spread, malignant tumors can migrate to other parts of the body. Cancers occur when genes are mutated and cell growth is abnormally regulated. Currently, oncogenes that cause cell cycle progression and contribute to cell proliferation, and tumor suppressor genes that participate in cell cycle arrest are subjects of active research. Typical examples are *ERBB2* encoding oncogene HER2/neu [1], and *TP53* encoding tumor suppressor gene p53 [2]. Recent studies have reported that genes not encoding a specific protein, such as long non-coding RNAs (lncRNAs) also affect the development of cancer.

LncRNAs are RNA transcripts that are longer than 200 nucleotides and are not translated into proteins. Many efforts have been made to identify the biological roles of lncRNAs in nuclei and cytoplasm. In nuclei, lncRNAs recruit chromatin-modifying enzymes and act as transcriptional guides. LncRNAs can also bind to mRNA and splicing factors and participate in splicing processes [3,4]. Other lncRNAs can bind to promoters of specific genes and facilitate or suppress their transcription. In addition, lncRNAs may also target RNA polymerase and transcription factors and regulate the transcriptions and expressions of genes [5]. For example, lncRNA YY1 directly interacts with transcription factor YY1 in muscle cells to activate gene expression by removing YY1 and polycomb repressive complex (PRC2) from the promoters of target genes [6]. In cytoplasm, lncRNAs may serve as competing endogenous RNAs (ceRNAs) that target microRNAs (miRNAs) [5], and interactions between lncRNA and miRNA targets, disrupts binding between the miRNA and its target mRNA, and leads to the induction of mRNA translation. In addition, lncRNAs can interact with proteins possessing RNA binding motifs and increase their stability and activity [7,8]. Such lncRNAs participate in gene regulation at the transcriptional and translational levels and in protein activation at the post-translational levels. Recently, several studies have reported that based on their biological activities, lncRNAs are highly associated with various diseases including cancer [9,10]. When cancer cells with counterpart normal cells were compared in various types of cancer, abnormal lncRNA expressions were observed in cancer cells. In addition, lncRNAs were found to regulate cell growth and proliferation during cancer development. Moreover, it was recently reported that long non-coding RNA linc00641 can sponge miR-424-5p in non-small cell lung cancer cells and act as a tumor suppressor [11].

Linc00152 (also known as cytoskeleton regulator RNA [CYTOR]) is an lncRNA located at 2p 11.2. Studies have shown that linc00152 is overexpressed in cancer cells and promotes cancer cell proliferation and metastasis. For example, it was found that the upregulation of linc00152 is associated with enhanced cell invasion in gastric adenocarcinoma cells [12]. In general, linc00152 is involved in the sponging of miRNAs or in the transcriptional silencing of tumor suppressor genes; activities that lead to cell proliferation and epithelial-mesenchymal transition. Here, we focus on the role of linc00152 during cancer development and summarize the findings of recent studies on the effects of linc00152 expression on cancer progression and on the molecular mechanisms of linc00152 in human cancers.

## Biological Functions of linc00152 in Cancer Cells

### Linc00152 acting as a ceRNA (miRNA sponging)

One of the roles of linc00152 is to function as a ceRNA of miRNA. The ceRNA activity of linc00152 may be associated with the upregulations of various counterpart mRNAs (originally targeted by miRNAs) involved in cell proliferation, survival, protection from apoptosis, invasion, and migration (Table 1). It has been reported that linc00152 might directly bind to miR-125b, up-regulate Mcl-1 (myeloid cell leukemia-1), and protect ovarian adenocarcinoma cells from apoptosis [13]. Linc00152 might also sponge miR-193a-3p, and thus, contribute to the upregulation of Mcl-1 expression [14]. In addition, linc00152 may promote cell proliferation and increase cell migration in gastric adenocarcinoma cells by sponging miR-193b-3p, and thus, upregulating ETS1 [15]. In another study, it was found that sponging of miR-139-5p by linc00152 up-regulated Notch1, which promoted cell proliferation, cell invasion, and migration in colorectal carcinoma cells [16]. Linc00152 might also promote cell proliferation by sponging miR-216b-5p, and thus, up-regulate the expression of homeobox A1 [17]. In addition, linc00152 might enhance the invasion and migration capacities of cancer cells by sponging miR-138 and upregulating hypoxia-inducible factor-1α (HIF-1α) expression [18]. Linc00152 may also interact directly with miR-139-5p, which is associated with the expressional upregulation of protein kinase AMP-activated catalytic subunit alpha 1, which is involved in the promotion of aerobic glycolysis for metabolic reprogramming [19]. These results suggest the associations between linc00152 and HIF-1α activation and increased aerobic glycolysis might be linked to cancer cell survival against intratumoral hypoxia, and contribute to tumor aggressiveness and malignant development. Several studies have reported that linc00152 might be involved in cell cycle regulation through its miRNA-sponging activities. In one study conducted using osteosarcoma cells, it was suggested that linc00152 transcriptionally activated by TCF3 (a transcription factor) might bind to miR-1182, up-regulate CDK14, and promote cell proliferation and migration [20]. In another study, linc00152 was found to up-regulate cyclin D1 and cyclin-dependent kinase 9 by sponging miR-193a [21,22]. Furthermore, it was reported that linc00152 increased the expressions of several proto-oncogenes by sponging their counterpart miRNAs in various types of cancer. It was suggested that linc00152 might down-regulate miR-612, be involved in the overexpression of Akt2, contribute to the activation of the nuclear factor kappa-light chain-enhancer of activated B cells (NF-κB) pathway, and consequently inhibit glioblastoma cell apoptosis [23]. In another study, results indicated that linc00152-dependent miR-153-3p down-regulation up-regulated Fyn (a proto-oncogene) and led to the induction of cell proliferation and the suppression of apoptosis in esophageal squamous cell carcinoma cells [24]. Thus, many studies have shown that linc00152 might be responsible for upregulations of several genes by direct binding miRNAs and disrupting their interactions with target mRNAs. Consequently, gene products up-regulated by the ceRNA activity of linc00152 may be involved in the promotions of cell proliferation, survival, and cell motility and in the suppression of apoptosis, which suggests linc00152 is a potential oncogenic ceRNA that promotes cancer progression.

### Linc00152 acting as a transcriptional regulator

**Fig. 1.**
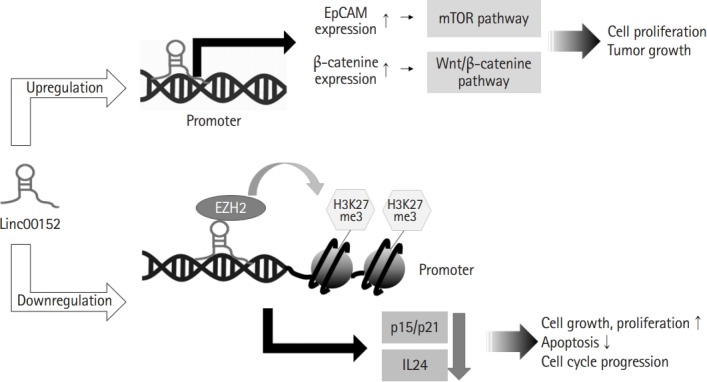
Linc00152 can participate in gene transcription. Linc00152 bound to gene promoters and up-regulated expression of epithelial adhesion molecule (EpCAM) and β-catenine, contributing to cell proliferation and cancer development through the activation of mammalian target of rapamycin (mTOR) and Wnt/β-catenine pathways, respectively. In addition, linc00152 recruited enhancer of zeste homolog 2 (EZH2) to the promoter of *p15, p21* and interleukin 24 (*IL24*) responsible for down-regulated expression of these genes, leading to promotion of cell proliferation and inhibition of apoptosis.

Linc00152 can bind to the promoter regions of specific genes and regulate their transcriptions (Fig. 1). One study presented that linc00152 can bind to the promoter of epithelial adhesion molecule (*EpCAM*) gene, and thus, induce cell proliferation and tumor growth by inducing the transcriptional upregulation of EpCAM and activation of the mammalian target of rapamycin pathway [25]. In addition, linc00152 may participate in the transcriptional repression of interleukin 24 (*IL24*) caused by the recruitment of enhancer of zeste homolog 2 (EZH2) to *IL24* promoter. EZH2 as a histone methyltransferase participate in trimethylation of histone H3 Lys 27 at the *IL24* promoter region, and thereby, facilitated lung adenocarcinoma cell growth [26]. Linc00152 also recruited EZH2 to *p15* and *p21* promoters, and thus, inhibited the transcriptions of p15 and p21, which increased cell cycle progression and cell proliferation [27]. In addition, linc00152 caused the proliferation of bladder carcinoma cells by upregulating β-catenin expression and directly activating the Wnt/β-catenin pathway [28]. Several studies on the roles of linc00152 in cancer cells have reported that linc00152 was involved in the transcriptional activations of oncogenes by directly binding to gene promoters and in the transcriptional repressions of tumor suppressors by interacting with other transcription factors. These findings indicate that linc00152 functions as an oncogenic transcriptional regulator.

### Linc00152 and regulation of protein activity

**Fig. 2.**
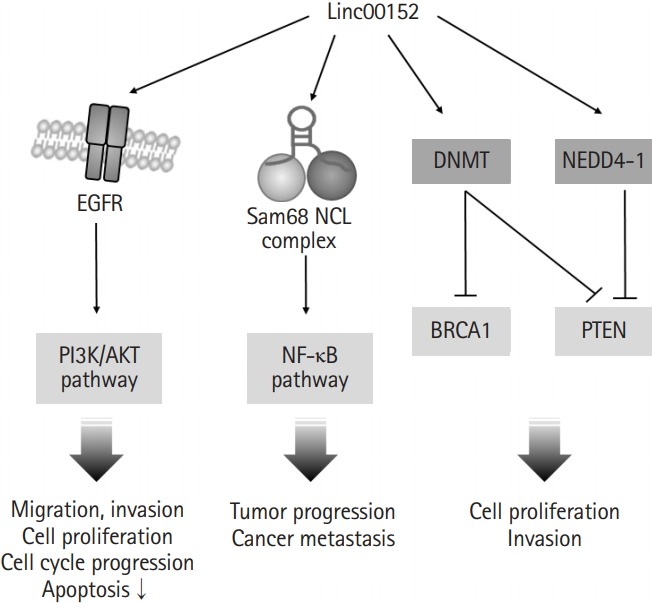
Linc00152 is involved in protein activation. Linc00152 bound directly to epidermal growth factor receptor (EGFR). The activated EGFR/phosphoinositide 3-kinase (PI3K)/AKT pathway promoted cell proliferation and migration. In addition, the complex of linc00152 and Sam68 promoted tumor progression through the activation of nuclear factor kappa-light chain-enhancer of activated B cells (NF-κB) pathway. DNA methyltransferase (DNMT) and NEDD4-1 (neural precursor cell expressed developmentally down-regulated protein 4-1) activated by linc00152 promoted cancer cell proliferation through inhibition of breast cancer type1 susceptibility protein (BRCA1) and phosphatase and tensin homolog (PTEN).

In addition to the involvement of linc00152 in transcription regulation via binding to the promoter regions of genes or miRNAs, linc00152 can also promote cell proliferation by directly binding to specific proteins and regulating their activities (Fig. 2). One study showed that linc00152 is capable of complex formation with two RNA binding proteins, NCL (nucleolin) and Sam68 (KHDRBS1, the src-associated substrate in mitosis of 68kDa), which are responsible for the development of colorectal cancer via NF-κB pathway activation [29]. Another study presented that linc00152 directly bound to epidermal growth factor receptor (EGFR) and increased the activity of EGFR to promote the EGFR/phosphoinositide 3-kinase/Akt pathway, which contributed to the inductions of tumorigenic features (e.g., cell cycle progression, cell proliferation, and migration) [30,31]. Moreover, linc00152 might be involved in the ubiquitin-dependent degradations of phosphatase and tensin homolog (PTEN) through the activation of NEDD4-1 (neural precursor cell expressed developmentally down-regulated protein 4-1) in breast adenocarcinoma [32]. It has also been reported that interaction between linc00152 and DNA methyltransferase (DNMT) may result in DNMT activation, the inhibitions of BRCA1 (breast cancer type 1 susceptibility protein) and PTEN, and increased cell proliferation and invasiveness in triple-negative breast adenocarcinoma and breast ductal carcinoma [33]. These studies show that proteins affected by linc00152 are involved in signaling pathways that contribute to cell survival, proliferation, and apoptosis, and suggest that linc00152 might facilitate malignant progression by activating cell survival/proliferation pathways and inhibiting of apoptotic pathways by interacting with specific proteins and by upregulating the transcriptional expressions of oncogenes.

## Clinical Implication of linc00152 Expression in Human Cancer

Several studies have addressed relations between linc00152 expression and malignant development. These studies revealed that linc00152 was overexpressed in various cancer types, including liver cancer, gastric cancer, lung cancer, and breast cancer [14,24,30,33]. Moreover, clinical studies have shown that high linc00152 expression in tumor tissues is associated with poor survival and disease-free survival [17,21,23,29,34]. Linc00152 expression has also been shown to be significantly correlated with tumor progression stage. Among cancer patients of TNM stage III or greater, the proportion of patients with high linc00152 expression was significantly higher than among patients with low linc00152 expression [13,16,25-27,31,35]. In a study of esophageal squamous cell carcinoma, patients with TNM stage I/II/III had higher linc00152 expressions [36]. Furthermore, linc00152 expression was also shown to be positively correlated with tumor size. Among patients with a tumor larger than 5 cm, the proportion of patients with high linc00152 expression was significantly greater than among patients with low expression [25-27,31,35], and in a study on osteosarcoma, a significantly greater proportion of patients with a tumor size of >3 cm exhibited ‘high’ linc00152 expression [20]. These results demonstrate that the expression of linc00152 is higher in tumor tissues than in normal tissues, that its expression is positively associated with tumor progression, and that high linc00152 expression is associated with poor prognosis in cancer patients. Furthermore, they indicate that linc00152 might act as a promising diagnostic and prognostic oncogene.

## Conclusion

Many studies have investigated the association between linc00152 and human cancers. Linc00152 expression has been shown to be greater in tumor tissues than in normal tissues and to function as an oncogene by promoting cell proliferation, tumor growth, and metastasis *in vivo*. Linc00152 acts on cancer cells in three major ways: by acting as a ceRNA and sponging miRNAs, by binding to promoter regions of specific genes to activate or inhibit transcription, or by directly binding to and regulating the activities of proteins. In cancers (e.g., ovarian, gastric, and colorectal cancer), linc00152 acts as a ceRNA and sponges various miRNAs, especially miR-125b, miR-193a, miR193b, miR-612, miR-138, miR-216b-5p, miR-153-3p, miR-1182 or miR-497, in cancer cells, and thus, increase the expressions of downstream genes targeted by miRNAs, and consequently, these increases promote cell proliferation, metastasis, and tumor development. Linc00152 might also participate in the transcriptional activations or repressions of specific genes (e.g., *EpCAM, IL24, p15*, and *p21*) and activate oncogenic signaling pathways by interacting with several proteins (e.g., EGFR and NCL/Sam68 complex). Accordingly, linc00152 facilitates cancer cell development by directly or indirectly controlling the expressions and activities of many genes involved in the cell cycle and cell proliferation. By integrating and analyzing the biological roles of linc00152 during cancer progression, linc00152 may prove to be a useful diagnostic and prognostic biomarker for human cancers.

## Figures and Tables

**Table 1.** Linc00152-sponged miRNAs and their target genes, biological consequences in cancer cells

Target miRNA	Target gene of miRNA	Biological consequences	Cancer type	Reference
miR-125b	Mcl-1	Inhibition of apoptosis	Ovarian adenocarcinoma	[13]
miR-193a-3p		Cell proliferation	Gastric adenocarcinoma	[14]
miR-193b-3p	ETS1	Cell proliferation and migration	Gastric adenocarcinoma	[15]
miR-139-5p	PRKAA1	Glycolytic phenotype	Gastric adenocarcinoma	[19]
	Notch1	Cell proliferation, cell invasion and migration	Colorectal carcinoma	[16]
miR-193a	CDK9	Cell cycle progression, cell proliferation, and inhibition of apoptosis	Acute monocytic leukemia, acute promyelocytic leukemia	[21]
miR-612	Akt2	Tumor growth, cell invasion, and inhibition of apoptosis	Glioblastoma	[23]
miR-138	HIF-1α	Cell invasion and migration	Gallbladder carcinoma	[18]
miR-216b-5p	HOXA1	Cell proliferation	Cervix epidermoid carcinoma	[17]
miR-153-3p	Fyn	Cell proliferation and inhibition of apoptosis	Esophageal squamous cell carcinoma	[24]
miR-1182	CDK14	Cell proliferation and migration	Osteosarcoma	[20]
